# Characterising extracellular vesicles from individual low volume cerebrospinal fluid samples, isolated by SmartSEC

**DOI:** 10.1002/jex2.55

**Published:** 2022-08-31

**Authors:** Yael Hirschberg, Kurt Boonen, Karin Schildermans, Annemieke van Dam, Isabel Pintelon, Charysse Vandendriessche, Milica Velimirovic, An Jacobs, Roosmarijn E. Vandenbroucke, Inge Nelissen, Yannick Vermeiren, Inge Mertens

**Affiliations:** ^1^ Health Unit Flemish Institute for Technological Research (VITO) Mol Belgium; ^2^ Centre for Proteomics (CfP) University of Antwerp Antwerp Belgium; ^3^ Biomedical Engineering and Physics Amsterdam UMC Amsterdam The Netherlands; ^4^ Department of Veterinary Sciences University of Antwerp Antwerp Belgium; ^5^ VIB Center for Inflammation Research VIB Ghent Belgium; ^6^ Department of Biomedical Molecular Biology Ghent University Ghent Belgium; ^7^ Department of Chemistry Atomic & Mass Spectrometry Ghent University Ghent Belgium; ^8^ Sustainable Chemistry Flemish Institute for Technological Research (VITO) Mol Belgium; ^9^ Faculty of Medicine & Health Sciences Translational Neurosciences University of Antwerp Antwerp Belgium; ^10^ Division of Human Nutrition and Health Chair group of Nutritional Biology Wageningen University & Research (WUR) Wageningen The Netherlands

**Keywords:** cerebrospinal fluid, extracellular vesicles, low sample volume, size exclusion chromatography

## Abstract

Extracellular vesicles (EVs) are suggested to have a role in the progression of neurodegeneration, and are able to transmit pathological proteins from one cell to another. One of the biofluids from which EVs can be isolated is cerebrospinal fluid (CSF). However, so far, few studies have been performed on small volumes of CSF. Since pooling of patient samples possibly leads to the loss of essential individual patient information, and CSF samples are precious, it is important to have efficient techniques for the isolation of EVs from smaller volumes. In this study, the SmartSEC HT isolation kit from System Biosciences has been evaluated for this purpose. The SmartSEC HT isolation kit was used for isolation of EVs from 500 μL starting volumes of CSF, resulting in two possible EV fractions of 500 μL. Both fractions were characterised and compared to one another using a whole range of characterisation techniques. Results indicated the presence of EVs in both fractions, albeit fraction 1 showed more reproducible results over the different characterisation methods. For example, CMG (CellMask Green membrane stain) fluorescence nanotracking analysis (NTA), ExoView, and the particles/μg ratio demonstrated a clear difference between fraction 1 and 2, where fraction 1 came out as the one where most EVs were eluted with the least contamination. In the other methods, this difference was less noticeable.

We successfully performed complementary characterisation tests using only 500 μL of CSF starting volume, and, conclude that fraction 1 consisted of sufficiently pure EVs for further biomarker studies. This means that future EV extractions may be based upon smaller CSF quantities, such as from individual patients. In that way, patient samples do not have to be pooled and individual patient information can be included in forthcoming studies, potentially linking EV content, size and distribution to individualised neurological diagnoses.

## 1 INTRODUCTION

Extracellular vesicles (EVs) are a heterogeneous group of nano‐sized particles that are secreted by cells into the extracellular space in both normal and pathological conditions. They can differ in size, morphology, composition – of, for example, protein and nucleic acid cargo – or biogenic mechanisms. Based upon these characteristics, three prominent subtypes of EVs have been described: exosomes, microvesicles and apoptopic bodies. The latter two generally have a diameter from 100 to 1000 nm and from 1 to 4 μm, respectively, and are formed by outward budding of the plasma membrane. In contrast, exosomes range from 30 to 150 nm and are formed by inward budding of the late endosome lumen to form a microvesicular body that is secreted by fusion with the plasma membrane, which was already shown in the 80s (HARDING et al., 1984; JOHNSTONE et al., 1987). The nomenclature of these particles was first discussed in 2013 by the *International Society for Extracellular Vesicles* (ISEV), a new organisation at the time (GOULD & RAPOSO, 2013), and then to a greater extent in 2014 (LOTVALL et al., 2014). New types of EVs are still being discovered, such as the recently observed exomeres (ZHANG et al., 2019) or supermeres (ZHANG et al., 2021).

**FIGURE 1:**
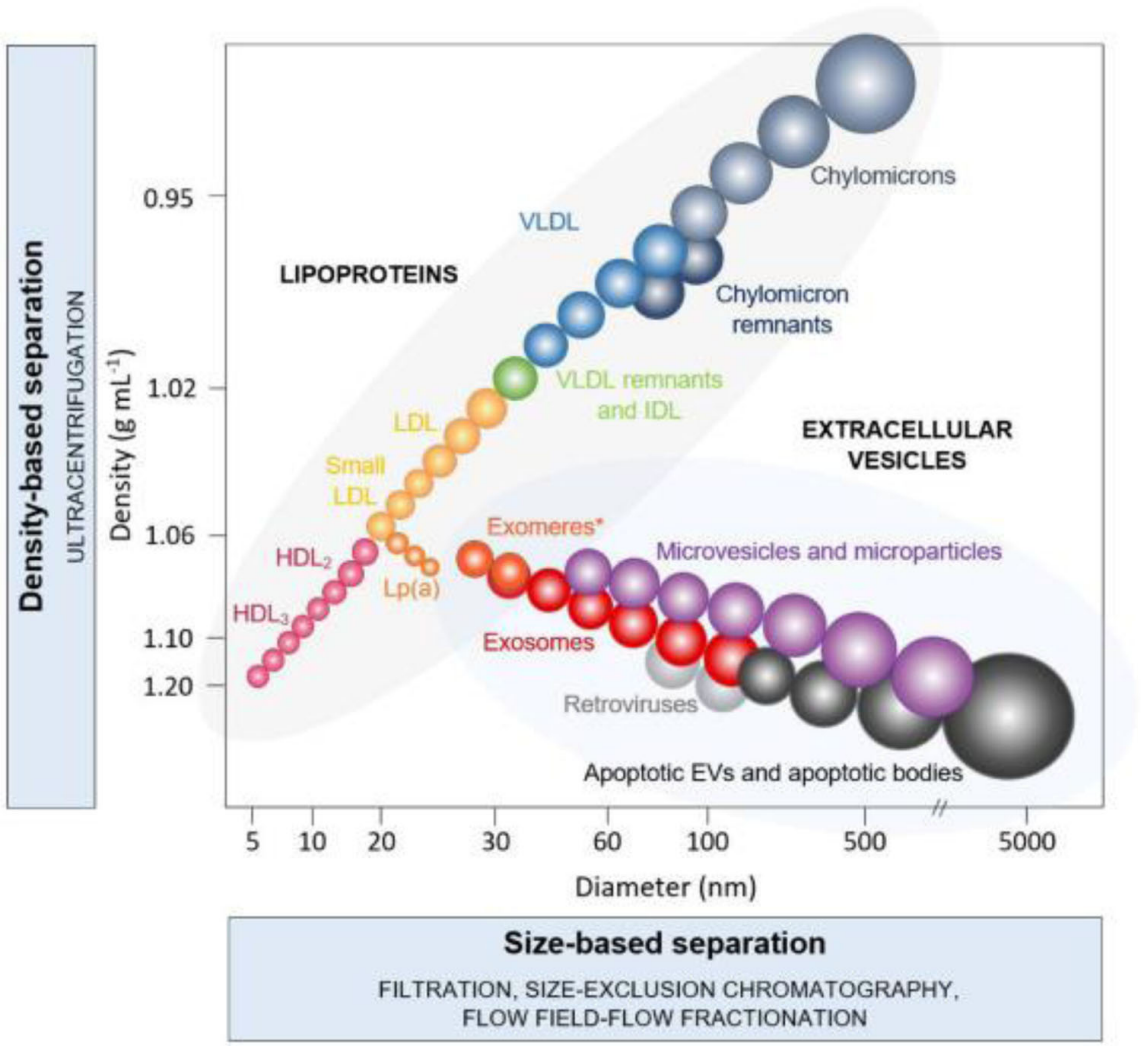
Subtypes of lipoproteins and extracellular vesicles (EVs) according to their density and size. HDL, high‐density lipoprotein; IDL, intermediate‐density lipoprotein; LDL, low‐density lipoprotein; Lp(a), lipoprotein(a); VDL, very‐low density lipoprotein. *The density of exomeres has not yet been determined experimentally. Reprinted from LIANGSUPREE et al. (2021), licensed under CC BY 4.0

The suggested roles of EVs in the central nervous system (CNS) are to dispose waste membrane and cellular materials, and to serve as messengers for communication between neural cells. In that regard, EVs can participate not only in the development of the CNS, but also in regulating synaptic activity, as well as regeneration following injury. Within the nervous system, neurons, oligodendrocytes and microglia secrete EVs that can be directed from one cell type to the other (KRAMER‐ALBERS et al., 2007; LACHENAL et al., 2011; MASYUK et al., 2010). Acting as intercellular communicators, EVs, more specifically exosomes, recently seized significant interest as potential diagnostic agents (VAN NIEL et al., 2018). The exploitation of EVs as a biomarker source benefits from separating EVs from non‐EV proteins and lipid particles, which remains difficult, especially while retrieving a high yield of particles. In Table 1, different biological structures that are in the same size range as EVs are described. The first description of lipoproteins in human cerebrospinal fluid (CSF) was published 60 years ago (SWAHN et al., 1961). Nevertheless, it is still a challenge to differ between lipoproteins and EVs. Lipoproteins consist of several major apoproteins in their membrane that can be used as markers, such as ApoB‐48, ApoE, ApoB‐100, ApoC, ApoA‐I and ApoA‐II. It should be noted that apolipoproteins are possibly also part of the cargo of EVs (NIKITIDOU et al., 2017). ApoE, for example, is also synthesised in the brain, where it is primarily produced by astrocytes (PITAS et al., 1987), but also by microglia (STONE et al., 1997), and by neurons (XU et al., 1999) under stress or damaged conditions. Figure 1 illustrates the comparison in density and size, both physical characteristics on which EV isolation methods are based, between multiple subtypes of lipoproteins and EVs (LIANGSUPREE et al., 2021).

**TABLE 1:** Biological structures and their nanoscale size, similar to exosomes

Structure	Size	Extracellular markers
**High‐density lipoprotein (HDL)**	7–14 nm (OTVOS, 2002)	ApoA‐I, ApoA‐II (SMITH et al., 1978)
**Low‐density lipoprotein (LDL)**	22–27 nm (JONAS, 2002)	ApoB‐100 (MAHLEY et al., 1984)
**Intermediate‐density lipoprotein (IDL)**	25–50 nm (GARRET & GRISHAM, 1995)	ApoB‐100, ApoE (BROWN & GOLDSTEIN, 1986)
**Ribosome**	30 nm (ALBERTS et al., 1994)	NTA
**Very‐low density lipoprotein (VLDL)**	30–90 nm (RENSEN et al., 2001)	ApoB‐100, ApoC‐I, ApoC‐II, ApoC‐III, ApoE (MAHLEY et al., 1984; SMITH et al., 1978)
**Supermere**	<50 nm (CLANCY et al., 2022)	HSPA13, ENO2, TGFBI, ENO1, GPC1 (ZHANG et al., 2021)
**Exomere**	<50 nm (CLANCY et al., 2022)	TGFBI, ENO1, GPC1 (ZHANG et al., 2021)
**Lysosome**	200–500 nm (ALBERTS et al., 1994)	NTA
**Chylomicron**	200–600 nm (JONAS, 2002)	ApoA‐I, ApoB‐48, ApoC‐I, ApoC‐II, ApoC‐III (MAHLEY et al., 1984; SMITH et al., 1978)

*Note* that for ribosomes and lysosomes, no extracellular markers are described as these are intracellular structures.

In the last decade, multiple isolation methods for EVs have been developed and commercialised. A world‐wide survey conducted in 2016 by Gardiner et al. (GARDINER et al., 2016) disclosed that ultracentrifugation remains the most commonly used isolation method. In this protocol, often a 10,000 × *g* centrifugation step is used in order to pellet larger vesicles. A study from 2012, however, showed that a certain amount of small EVs is also pelleted at this step (BOBRIE et al., 2012). Next, reproducibility seems difficult to be achieved as the centrifugation rotor type and time have an influence on yield and purity of the EVs (CVJETKOVIC et al., 2014). Repeated centrifugation steps are thus often required to reduce the amount of co‐isolation with non‐EV particles. However, this results in reduced yield due to damaged and lost EVs. Another similar technique is to separate and purify EVs based on their buoyant density by the use of a density gradient medium such as iodixanol or sucrose, followed by centrifugation. The primary step can cause co‐isolation of non‐EV particles of a similar density. Here, the centrifugation step, in contrast to differential centrifugation EV isolation, prevents pelleting of the EVs and thus avoids loss and damage of EVs. Co‐precipitation of other particles is also observed with polymer‐based approaches, which thus show high yield but low purity. For classical size exclusion chromatography (SEC), the main disadvantage is the dilution of EVs in multiple fractions, often requiring an additional concentration step after EV collection (BRENNAN et al., 2020; PATEL et al., 2019; SIDHOM et al., 2020). Other possible isolation methods include ultrafiltration, flow‐field flow fractionation or immunoprecipitation (BRENNAN et al., 2020; KONOSHENKO et al., 2018; LIANGSUPREE et al., 2021), or novel isolation methods such as EXODUS (CHEN et al., 2021)

To date, there is no consensus on each method's suitability for clinical and scientific applications as each method has its drawbacks (BUSCHMANN et al., 2018). Techniques other than the “golden standard” method ultracentrifugation have gained preference (GARDINER et al., 2016) when sample starting volume is limited. Starting volume mainly depends on the availability and accessibility of the biofluid used. Lumbar puncture, for example, is a relatively invasive biofluid extraction technique, and, therefore, CSF samples are precious with limited availability. Nonetheless, for diseases of the CNS, to date, CSF is still the most used biofluid since it reflects overall brain biochemistry. Enriching CSF for EVs can help reduce the concentration of abundant non‐relevant proteins, and in turn concentrate potential neurological disease biomarkers (WELTON et al., 2017). In Table 2, an overview is provided of human CSF EV isolation methods applied in previous studies, focusing on AD, as well as multiple sclerosis or amyotrophic lateral sclerosis. Studies where RNA was extracted during the main isolation method were excluded from this table as proteins were removed in these methods and these are thus not relevant to proteomic research.

**TABLE 2:** Overview of EV isolation methods previously described

Method	Starting volume of human CSF	Method	Starting volume of human CSF
**Differential centrifugation**	200–500 ml (STREET et al., 2012) 2.5 ml (STUENDL et al., 2016) 2.5 ml (LEE et al., 2016) 2 ml (WANG et al., 2017) 1 ml (AKERS et al., 2017) 10 ml (GUIX et al., 2018)	Ultrafiltration and size exclusion chromatography (EVSecond)	3.5 ml (HAYASHI et al., 2020)
**Size exclusion chromatography**	500 μL–3 ml (NORMAN et al., 2021; KRUSIC ALIC et al., 2022)	Ultrafiltration and liquid chromatography	7.2 ml (THOMPSON et al., 2020)
**CD11b Immunocapture**	3 ml (GUO et al., 2020)	Immunodepletion and ExoQuick	400 μL (GUHA et al., 2019)
**MagCapture Exosome Isolation Kit PS**	1–4 ml (MURAOKA et al., 2019; MURAOKA et al., 2020)	Ultracentrifugation and ExoQuick	5 ml (MINAKAKI et al., 2018)
**Total Exosome Isolation Kit/miRCURY Exosome Isolation Kit**	1 ml (LOPEZ‐PEREZ et al., 2021) 2–4 ml (LEE et al., 2021)	Ultracentrifugation and sucrose gradient fractionation	1–4 ml (SAMAN et al., 2012) 4–6 ml (CHIASSERINI et al., 2014)
**Exo‐Spin**	5 ml (WELTON et al., 2017)	Ultracentrifugation and ultrafiltration	1 ml (MANEK et al., 2018)
**EXODUS**	2 ml (LI et al., 2021)		
**FACS**	100 μL (PIERAGOSTINO et al., 2019)		

Only those studies that used human CSF were included. Alike studies that extracted RNA were excluded from this table, since proteins are removed with these methods.

In most of these studies, 1–5‐ml CSF is used, which often necessitates a pool of patient samples (e.g. in LEE et al. (2016)). In contrast, in a clinical setting with hundreds of samples, pooling is out of the question given the patients’ diagnoses based upon individual CSF biomarker assessments. Other methods, like affinity isolation methods such as CD11b immunocapturing (where only the microglia/macrophage‐derived EVs are researched) (GUO et al., 2020), exclude a certain group of EVs. Another affinity‐based approach is the MagCapture Exosome Isolation Kit, based on pulling down phosphatidylserine. In PIERAGOSTINO et al. (2019), a small volume of CSF was used for FACS isolation, but here the size measured of these EVs was between 333 and 1326 nm, excluding the small EVs. In the last year, also SEC has been performed on CSF (KRUSIC ALIC et al., 2022; NORMAN et al., 2021). However, in these papers, there is a lack of validation of the technique starting with small sample sizes.

**FIGURE 2:**
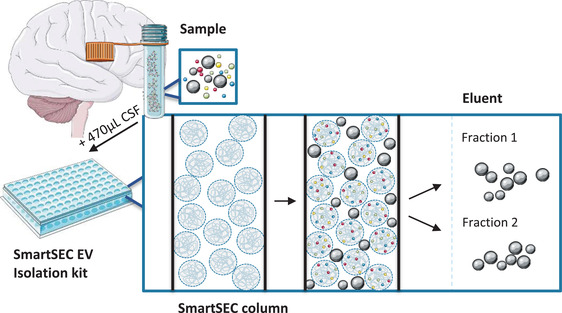
Illustration of the SmartSEC EV isolation technique. Particles that can pass the size exclusion columns are collected in the eluent, while small proteins (up to 400 kDa) enter the bead core and, because of affinity interaction modes, stay trapped. Figure adapted from System Biosciences, with figures from smart.servier.com (licensed under CC BY 3.0)

In the current study, we aim to assess the novel SEC kit called SmartSEC HT EV Isolation System (System Biosciences, USA) for isolation of EVs from CSF. This kit provides an EV isolation system in 96‐well format for high‐throughput (HT). It is optimised for serum and plasma, and the isolation of CSF EVs was not evaluated by System Biosciences. In less than 1 h, two subsequently eluted EV fractions are collected. With this technique, size exclusion and affinity interaction modes are combined (see Figure 2). SmartSEC columns are filled with porous beads, of which the inside core is functionalised with proprietary affinity interacting modes enabling the capture and retention of protein impurities up to 400 kDa (15–20 nm) (as described in the product specification file). For characterisation of the SmartSEC EV fractions, various complementary techniques were used, as advised by the guidelines of the ISEV (THERY et al., 2018; Witwer et al 2021). By use of transmission electron microscopy (TEM), asymmetrical‐flow field‐flow fractionation (AF4) coupled with a multi‐angle light‐scattering (MALS) detector, and nanotracking analysis (NTA), the size and concentration of particles in the EV fractions were measured. The presence of EV‐specific markers was established by ExoView, surface plasmon resonance imaging (SPRi), and liquid chromatography tandem mass spectrometry (LC‐MS/MS).

## 2 MATERIAL AND METHODS

### 2.1 CSF collection

CSF for Alzheimer's disease and dementia with Lewy bodies (DLB) differential biomarker analyses was originally obtained from (non‐) dementia subjects (DLB, prodromal DLB, AD, prodromal AD and healthy controls) by means of lumbar puncture as described previously (ALCOLEA et al., 2014; ALCOLEA et al., 2015; MORENAS‐RODRIGUEZ et al., 2019). All subjects signed the informed consent to participate in the study. CSF was collected and processed in polypropylene tubes following international recommendations (DEL CAMPO et al., 2012). The first 2 ml was excluded, the next 10 ml was centrifuged (2000 × *g* at 4°C for 10 min) to avoid any possible hematic contamination and aliquoted within the first 2 h after the lumbar puncture. Aliquots were stored at −80°C until further analysis as described by MORENAS‐RODRIGUEZ et al. (2019) UHPLC‐ECD was also performed on one freshly frozen CSF aliquot of 200 subjects out of the abovementioned study at the University of Antwerp‐Institute Born‐Bunge (UA‐IBB) for neurotransmitter analysis (unpublished results). The CSF remainders of the 200 subjects were pooled following UHPLC‐ECD and subsequently provided by the Neurobiobank of IBB (Wilrijk, Antwerp; BB190113) upon formalised request, solely for the purpose of EV isolation experiments. From this pool, 500 μL CSF aliquots were prepared for further processing as described below.

Each newly composed 500 μL aliquot contained CSF that underwent two previous freeze‐thaw cycles, that is one prior to UHPLC‐ECD, and, one whilst preparing the aliquots from the CSF remainders.

### 2.2 Extracellular vesicle isolation

The newly composed 500 μL CSF aliquots were thawed on ice and centrifuged 10 min at 2500 × *g* to remove cell debris prior to EV isolation using the SmartSEC HT kit (System Biosciences, USA) following manufacturer's instructions: After preparing the SmartSEC filter plate, 470 μL of sample was added to the wells. After an incubation step of 30 min at room temperature, the first fraction was collected by centrifuging the plate for 2 min at 500 × *g*. All centrifugation steps in this study were performed with a centrifuge 5424 R and a fixed angle rotor. To collect the second fraction, 470 μL of SmartSEC HT isolation buffer was added to each well and the plate was centrifuged again for 2 min at 500 × *g*. The output volume is about as much as the input volume, in this case about 470 μL for both fractions collected. Until further use, samples were stored at −80°C. For all experiments described below, the samples were collected in the same way and with the same CSF pool.

### 2.3 Transmission electron microscopy (TEM)

To visualise the EVs and characterise their morphology and size, TEM was performed. Samples were thawed at ambient temperature and vortexed for 10 s. Sample preparation was adapted from OEYEN et al. (2018). Per sample, three droplets of 20 μL were placed on a clean Parafilm, after which a nickel TEM grid was placed on top of each droplet for 60 min, which made it possible to adsorb the fluid. The grids with adherent EVs were washed three times with PBS for 2 min and five times with ultrapure water for 2 min. Subsequently, the droplets were fixed with 2.5% glutaraldehyde for 10 min, and washed five times with ultrapure water for 2 min. The grids were then transferred to 2% uranyl acetate to contrast for 15 min. Last, the grids were incubated in 0.13% methyl cellulose and 0.4% uranyl acetate and dried at room temperature before examination with Tecnai G2 Spirit BioTWIN (FEI, Eindhoven, The Netherlands). All solutions were prepared in ultrapure water and filtered (Millipore) and ultrapure water was heated to remove the CO_2_.

### 2.4 Asymmetrical‐flow field‐flow fractionation (AF4) coupled with UV–VIS and a multi‐angle light‐scattering (MALS) detector

The AF4 experiments protocol was adapted from SITAR et al. (2015) and OEYEN et al. (2018). For this protocol, tests were performed with 100, 200 and 900 μL of fraction 1 and fraction 2. To obtain the sample volume of 900 μL (maximum injection volume for sample loop), two fraction 1 samples as well as two fraction 2 samples were combined. EV fractionation and particle sizing were carried out at room temperature (20°C –25°C) using an Eclipse Dualtec AF4 system (Wyatt Technology, Dernbach, Germany) and an Agilent 1260 high‐performance liquid chromatograph (HPLC) equipped with a quaternary pump with integrated degasser and vial sampler (Agilent Technologies, USA). A UV–VIS detector at 280 nm (Agilent Technologies, USA, 1260 series) and 18‐angle MALS detector (DAWN Helios II, Wyatt Technology, USA) using a laser at 658 nm was coupled to the fractionation system. A pre‐cut semipermeable 10 kDa regenerated cellulose membrane (Millipore PLGC Membrane, pre‐cut purchased via Wyatt) and a 350 μm height spacer were introduced inside the small channel. The MALS detector was calibrated using anhydrous toluene (99.8% purity) (Sigma Aldrich, St. Louis USA) and normalised with an isotropic scatter standard. In this case, bovine serum albumin (BSA; Thermo Scientific) was used at a concentration of 1 mg/ml according to recommendations from Wyatt Technology Europe. The carrier solution consisted of a 1 x PBS (Gibco®, pH 7.4) filtered before use (Anodisc < 0.02 μm filter, Whatman, Maidstone, UK). For separation, the cross‐flow was maintained at 3 ml/min for 5 min followed by a 45 min exponential gradient type 5 to a cross‐flow of 0.05 ml/min. Other detailed separation settings for the AF4 experiments are summarised in Table 3.

**TABLE 3:** Experimental parameters for AF4

AF^4^	Unit	Value
**Tip to tip chancel length**	[cm]	15.4
**Focus flow rate**	[ml/min]	1.50
**Injection flow**	[ml/min]	0.2
**Injection time**	[min]	9
**Focus time**	[min]	1
**Elution time**	[min]	62
**Detector flow rate**	[ml/min]	0.5

The duration of a representative AF4 run was 75 min. The data acquisition interval was set to 1 s. Data were collected and analysed in Astra 6.1.7.17 software (Wyatt Technology, Santa Barbara, USA). The size of observed particles was expressed by the root mean square (rms) radius (*R*_rms_) calculated from data collected by 18 different angles from the MALS detector. The Zimm first‐order model was used to convert shown data to *R*_rms_ as previously reported by KAMMER et al. (2005).

### 2.5 ExoView

ExoView technology was applied using the ExoView Tetraspanin kit (EV‐TETRA‐C, NanoView Biosciences), which includes the chips and Solution A and B. EV fractions were diluted 1:2 and non‐purified CSF 1:30 using Solution A. Thirty‐five microlitres of the diluted sample were incubated on the chip (coated with CD9, CD81, CD63 and IgG control antibodies) overnight at RT in an aluminium‐sealed 24‐well plate without agitation. After four washes under shaking conditions with Solution A, chips were subjected to staining using anti‐CD81 CF555, anti‐CD9 CF488, anti‐CD63 CF647 (each antibody diluted 1:1200 in blocking solution) for 1 h without shaking. Subsequently, chips were washed seven times under shaking conditions, twice with Solution A, three times with Solution B and twice in ultrapure water. Lastly, the chips were dried, scanned by the ExoView R100 reader (NanoView Biosciences Boston, USA) and eventually analysed using the NanoViewer analysis software version 3.0.

### 2.6 Surface plasmon resonance imaging (SPRi)

Fractions 1 and 2 were loaded on 100 kDa Amicon filters and resuspended in 470 μL PBS. SPRi measurements were performed using the MX96 SPRi device (IBIS technologies B.V., Enschede, the Netherlands) as described by RIKKERT et al. (2020). Markers (5 μg/ml of albumin, apolipoprotein E (ApoE), caveolin‐1, CD9, CD81, CD63, flotillin‐1 (FLOT1), HSP70, lactadherin and tumour susceptibility gene 101 protein (TSG101) (see Table 4)) were printed in triplicate on a sensor surface (Easy2Spot type‐G; Ssens B.V., Enschede, The Netherlands) using a Continuous Flow Micro‐spotter (CFM) 2.0 (Wasatch microfluidics LLC, Salt Lake City, UT). Three spots were used as control spots, and active sites on the sensor surface were inactivated.

**TABLE 4:** Overview of the markers used for the surface plasmon resonance imaging experiment

Biomarker	Manufacturer	Product number
**Albumin (Alb (1))**	LSBio	LS‐C51819
**Albumin (Alb (2))**	Santa Cruz	SC271604
**Apolipoprotein E (ApoE)**	Novus Biologicals	NB400‐158
**Caveolin 1**	BD transduction	610406
**CD9**	BD pharmingen	555370
**CD63**	BD pharmingen	556019
**CD81**	BD Pharmingen	555675
**Flotilin‐1 (FLOT1)**	BD transduction	610821
**HSP70**	BD transduction	610607
**Lactadherin**	Haemtech	BLAX‐FITC
**TSG101**	Genetex	GTX70255
**Isotype control (IgG1)**	R&D systems	Mab002

Before starting the runs, three regeneration runs were applied, consisting of (i) a baseline phase of 2 min, (ii) an association phase of 15 min, whereby regeneration buffer (0.1 M Glycine (Merck, Darmstad, Germany), pH 3.06 and 0.3% (v/v) Triton‐x100 (Sigma‐Aldrich, Darmstadt, Germany)) is flowed back and forth over the sensor surface and (iii) a dissociation phase of 2 min, whereby the regeneration buffer is replaced by PBS. Next, 300 μL of each pre‐diluted sample was placed into the SPRi to allow measurement of each sample in duplicate. A single sample run consisted of (i) a baseline phase of 2 min, (ii) an association phase of 60 min, whereby the sample is flowed back and forth over the sensor surface and (iii) a dissociation phase of 4 min. After each sample run, the chip surface was regenerated to break the formed antigen‐antibody bonds before the next sample run was started. The sample was diluted 1:5 in PBS, so 30 μL of CSF EVs were analysed per sample run.

File conversion and analysis were performed as described before (RIKKERT et al., 2020; STAVERS et al., 2019). The first 100 s of the association phase were removed from the analysis to account for RI differences of buffers. The mean SPRi signals of each marker of the last 50 s of the association phase, corrected for the reference spot, were used as the maximum response. The difference between the maximum response and the response after 100 s of each spot was used to calculate the mean response in resonance units (RU). A data point represents the RI change during 1 h per sample averaged over the two spots. For normalisation, the lowest and highest RU values of each sample were set to 0% and 100%, respectively (with all other values in between).

### 2.7 Nanotracking analysis (NTA)

Particle concentration and size were determined using NTA, with a ZetaView instrument (Particle Metrix, Germany) in both scatter and fluorescence mode (488‐nm laser, 40 mW power), where EVs were bound to CMG (CellMask Green Plasma Membrane Stain, Thermo Scientific). Before measurement, samples were mixed with CMG (1/100 in filtered PBS, Gibco DPBS powder, no calcium, no magnesium, Fisher Scientific, filtered by a sterile 0.1 μm pore syringe filter, Millipore) and after a 1‐h incubation step in the dark at room temperature, EVs with CMG were diluted 1:2 with filtered PBS (sterile 0.1 μm pore syringe filter, Millipore) (final dilution CMG 1:80,000). This is the minimally required dilution since a total volume of at least 800 μL has to be injected in the instrument. Samples that demonstrated less than 25 particles per frame were excluded.

In fluorescence mode, only particles emitting fluorescence (normally at a larger wavelength compared to excitation) are detected, as the scattering light (with the same wavelength as the laser) is blocked by a filter with a certain cutoff wavelength (in this case 500 nm, 12 nm larger than the laser wavelength). This filter also blocks some of the fluorescence signal, which is why, prior to analysis, the system was verified with both 100‐nm polystyrene latex microbeads (Particle Metrix). FITC‐labelled silica beads (Nanocs Inc.) were used to determine the concentration correction factor by comparing the number of measured particles, taking the sensitivity into account, between scatter and fluorescence mode. These beads were used because of their similar scattering characteristics and refractive index. This resulted in a correction factor of 1.3, meaning the number of particles were underestimated in fluorescence mode by 23% compared to scatter mode.

For each measurement, two cycles of each 11 positions were performed, with a frame rate of 30. Settings were kept constant for all samples (focus: autofocus; camera sensitivity: 80.0 (scatter mode) or 97 (fluorescence mode); shutter: 100.0; temperature: 22.0°C). The videos were analysed by the ZetaView software version 8.05.11 SP2 (minimum brightness: 30, minimum particle size: 10, maximum particle size: 1000).

### 2.8 Protein concentration

Total protein content was determined using the NanoOrange Protein Quantitation Kit (Thermo Scientific) following the manufacturer's specifications. A standard curve of serially diluted BSA (Thermo Scientific) was used. Non‐purified CSF was diluted 1:100 in the assay. EV samples were vacuum‐dried and re‐solved in filtered PBS in a volume 1:3 of the dried‐down volume, and were diluted 1:50 as a final assay concentration. Values were extrapolated from the BSA curve, using a linear equation, with *r* ^2^ > 0.98.

### 2.9 Mass spectrometry

#### 2.9.1 Protein extraction from EV fractions

The sample preparation protocol was adapted from OEYEN et al. (2018). To perform the mass spectrometry sample preparation, 0.7 μg of protein was taken from samples 1–3, for sample four 1.0 μg was taken, measured with NanoOrange protein quantification.

SmartSEC fractions were vacuum‐dried in 2 ml protein LoBind tubes (Eppendorf, Germany) to obtain an almost‐dry pellet of EVs. A methyl tert‐butyl ether (MTBE) lipid extraction method was applied on the samples, based on the procedure described by MATYASH et al. (2008). To dissolve all of the hydrophilic molecules, the pellet was suspended in 300 μL methanol. After vortexing for 10 s, 1 ml of MTBE (Sigma, Belgium) was added to dissolve the hydrophobic molecules, and samples were shaken at 80 rpm for 1 h at room temperature. For phase separation, 260 μL water was added, followed by a 10 min incubation step at room temperature. After centrifugation for 10 min at 1000 × *g*, a lower hydrophilic with a protein layer on the bottom and an upper lipophilic phase appear. The first millilitre of the upper lipophilic phase was stored for lipidomic analysis. The lower hydrophilic layer was vacuum‐dried and used for proteomic analyses.

After resuspending this layer in 75 μL 5 M urea, samples were vortexed and sonicated for 10 min. Proteins were reduced in a final concentration of 10 mM dithiothreitol at 60°C for 30 min. Proteins were alkylated in a final concentration of 20 mM iodoacetamide in the dark at room temperature for 30 min. Overnight acetone precipitation was performed at −20°C, followed by centrifugation step (15,000 × *g* for 10 min) and decanting the supernatants. The pellet was resolved in 50 mM TEAB. To digest the proteins, 1 μg trypsin was added per 40 μg of protein and digestion was carried overnight at 37°C. Digests were desalted using Pierce C18 spin columns (Thermo Scientific, United States of America) according to manufacturer's instructions.

#### 2.9.2 Liquid chromatography tandem mass spectrometry (LC‐MS/MS) analysis

The peptide sample was dissolved in 10 μL of 6% acetonitrile (ACN) and 0.1% formic acid (FA) and separated on a ACQUITY UPLC M‐Class System (Waters), fitted with a nanoEase^TM^ M/Z Symmetry C18 trap column (100 Å, 5 μm, 180 μm × 20 mm) and a nanoEase^TM^ M/Z HSS C18 T3 Column (100 Å, 1.8 μm, 75 μm × 250 mm, both from Waters). The sample was loaded onto the trap column in 2 min at 5 μL/min in 94% buffer A 6% buffer B (buffer A is 0.1% FA in MilliQ, buffer B 0.1% FA in 80% ACN). The flow over the analytical column was 0.4 μL/min and the column was heated to 40°C. After an isocratic flow of 4 min at 6% B, the concentration of B increased in 120 min to 50% B, and then to 94% B in 4 min. Again after 4 min at 94% B, the concentration of B decreased in 4 min to 6%, which was followed by 14 min of equilibration at 6%.

The column was online with a timsTOF Pro operating in positive ion mode, coupled with a CaptiveSpray ion source (both from Bruker Daltonics GmbH, Bremen). The timsTOF Pro was calibrated according to the manufacturer's guidelines. The temperature of the ion transfer capillary was 180°C. The Parallel Accumulation–Serial Fragmentation (PASEF) DDA method was used to select precursor ions for fragmentation with 1 TIMS‐MS scan and 10 PASEF MS/MS scans, as described by MEIER et al. (2018). The TIMS‐MS survey scan was acquired between 0.70–1.45 V.s/cm^2^ and 100–1700 m/z with a ramp time of 100 ms. The 10 PASEF scans contained on average 12 MS/MS scans per PASEF scan with a collision energy of 10 eV. Precursor ions with 1 – 5 charges were selected with the target value set to 20,000 a.u and intensity threshold to 2500 a.u. Precursors were dynamically excluded for 0.4 s. The timsTOF Pro was controlled by the OtofControl 5.1 software (Bruker Daltonik GmbH). Ten PASEF scans contained on average 12 MS/MS scans per PASEF scan. Raw data were analysed with the DataAnalysis 5.1 software (Bruker Daltonik).

#### 2.9.3 MaxQuant

MaxQuant 1.6.7.0 software (Max‐Planck Institute, Germany) (COX & MANN, 2008) was used to perform database searches against the database Uniprot Human (Proteome ID: UP000005640, downloaded on 30th January 2020). During the search, the trypsin digest was allowed to have two missed cleavage sites. Carbamidomethyl modifications were defined as fixed modifications and acetylation of the N‐term and oxidation of methionine were variable modifications. Identifications were transferred between runs with the match‐between‐runs setting, and the LFQ (label‐free quantification) quantification method was used to calculate protein intensities.

#### 2.9.4 GO enrichment

DAVID Bioinformatics Resources 6.8 (https://david.ncifcrf.gov/tools.jsp) was used for the GO enrichment. The whole proteome background was retrieved from the database Uniprot Human (Proteome ID: UP000005640, downloaded on 30th January 2020), the CSF background was retrieved from MACRON et al. (2020), resulting in 3174 proteins.

## 3 RESULTS

### 3.1 Transmission electron microscopy (TEM)

**FIGURE 3:**
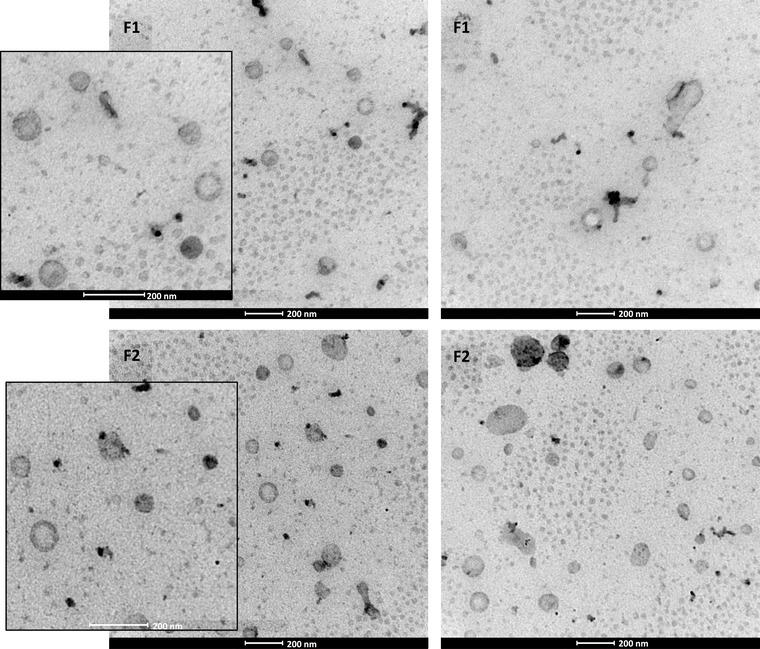
Transmission electron microscopy images of SmartSEC EV fraction 1 (F1) and fraction 2 (F2). Scale bars are 200 nm

In Figure 3, two TEM images of one fraction 1 sample and one fraction 2 sample are depicted. Both fractions were comparable in size and distribution. Particles with a double lipid membrane were observed in the size range of 90–110 nm, indicating the presence of small EVs. Also smaller particles of 20–50 nm could be observed, which could be low‐density lipoproteins (LDLs) or exomeres, and few larger particles of around 200 nm.

To exclude that these particles were coming from the SmartSEC buffer used during isolation, the buffer was also imaged on TEM. In the buffer, no such particles were observed (data not shown).

### 3.2 Asymmetrical‐flow field‐flow fractionation (AF4) coupled to UV–VIS and multi‐angle light‐scattering (MALS)

**FIGURE 4:**
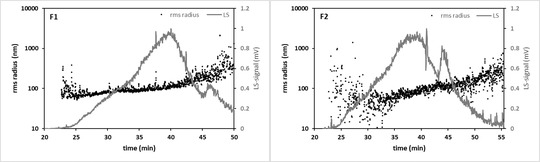
Plots of light scattering (LS) 90° response (dark grey) together with Rrms plots (black) versus elution time for fraction 1 (F1) and fraction 2 (F2)

AF4 can serve as a powerful platform for the characterisation of EVs (ZHANG & LYDEN, 2019) and consequently validation of the SmartSEC EV kit used for EV isolation from CSF. Combination of AF4 with UV–VIS and MALS measurements is critical for accurate size determination of particles and was used as a complementary technique to assess the novel EV isolation method, as well as the purity of both EV fractions. For 100 and 200 μL samples, no LS signal could be observed, while 900 μL, requiring the combination of two samples, clearly showed the presence of particles (Figure 4). For the maximum sample injection volume of 900 μL, particles eluted from 22 to 50 min for fraction 1 and 22 to 55 min for fraction 2. The *R*_rms_ (rms radius) of EVs was assessed using AF4‐MALS by fitting the recorded light scattering intensities to a particle scattering function and using Zimm data processing algorithm. Determined *R*_rms_ for fraction 1 (*R*_rms_ = 108.6 ± 1.0 nm) and fraction 2 (*R*_rms_ = 79.5 ± 1.2 nm) shows that fraction 1 compromises larger particles (median particle size of 217.2 nm) than those present in fraction 2 (median particle size of 159.0 nm). In fraction 2, a more diffuse pattern of sizes was observed.

In none of the samples, a signal could be retrieved by UV. It is suspected that abundance was not high enough to reach the detection limit, since this detector is less sensitive compared to the MALS detector. The UV signal being undetectable does suggest that there is not a lot of protein contamination in the fractions present. However, the purity of both EV fractions could not be compared by AF4‐UV‐MALS.

### 3.3 ExoView

**FIGURE 5:**
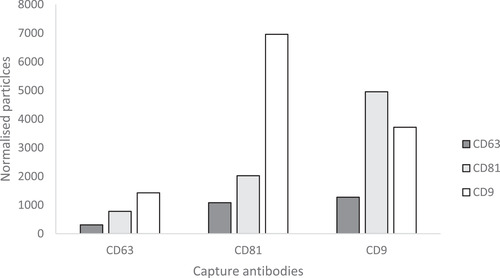
Illustration of tetraspanin positive extracellular vesicles in unpurified cerebrospinal fluid. Measured CD9‐, CD63‐ and CD81‐positive EVs (measured by fluorescence) are shown for CD63 captured particles, CD81 captured particles and CD9 captured particles

**FIGURE 6:**
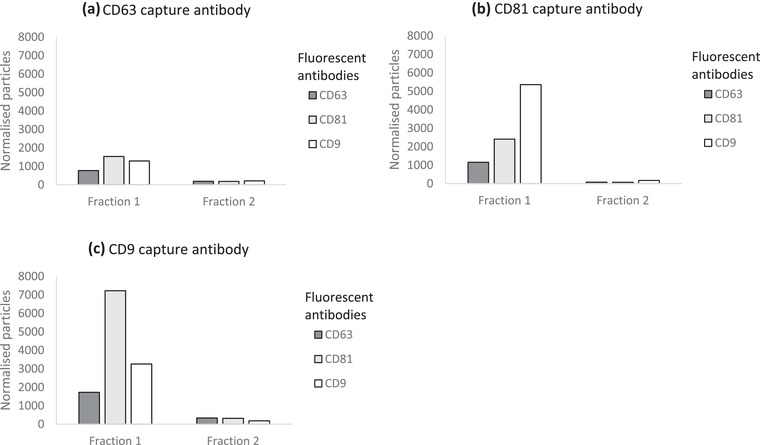
ExoView data comparison between fractions 1 and 2 eluted by the SmartSEC EV isolation kit. Measured CD9‐, CD63‐ and CD81‐positive EVs (measured by fluorescence) are shown for (a) CD63 captured particles (b) CD81 captured particles (c) CD9 captured particles

The amount of CD9‐, CD63‐ and CD81‐positive EVs in both non‐purified CSF and SmartSEC EV fractions 1 and 2 were determined by ExoView analyses. In Figures 5 and 6, respectively, the number of normalised particles are shown in both unpurified CSF and isolated EV fractions. CD63, CD81 and CD9 were used as capture and fluorescent antibodies. For unpurified CSF, an 1:30 dilution was used, for EV fractions 1 and 2 a dilution of 1:2. These dilutions were not taken into account in Figures 5 and 6, since some channels were saturated (>6000 particles on a chip).

ExoView analysis showed the presence of EVs in both unpurified CSF (Figure 5) and CSF EV fraction 1 (Figure 6). In CSF EV fraction 2 (Figure 6), little EVs could be observed by measuring tetraspanins CD63, CD9 and CD81. As was also the case for crude CSF, capture antibodies CD81 and CD9 resulted in the highest signal of the three fluorescent antibodies used. Furthermore, a trade‐off can be observed where the CD9‐captured EVs show a higher CD81 signal and the other way around. This could mean that most of the isolated EV in fraction 1 only have one epitope per particle. Another possible explanation is that fraction 2 consists of even smaller EVs, causing steric hindrance for two antibodies to bind on one particle.

### 3.4 Surface plasmon resonance imaging (SPRi)

**FIGURE 7:**
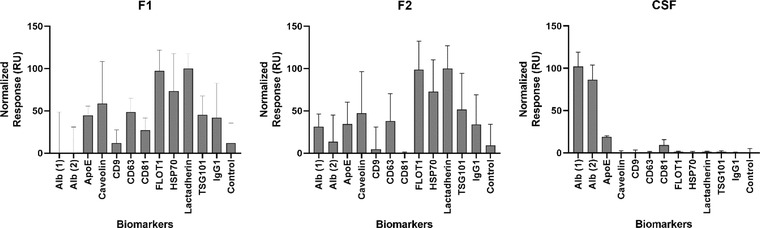
Surface plasmon resonance imaging (SPRi) response for different extracellular vesicle‐specific markers and IgG1. Samples used were fraction 1 (F1), fraction 2 (F2) and crude cerebrospinal fluid (CSF). The bars represent the mean value and SD of the technical replicates (*n* = 3)

A panel of nine EV markers was selected (TSG101, Lactadherin, caveolin, CD63, Flotillin‐1, HSP70, CD81 and CD9) and two contaminant markers (albumin, ApoE). Figure 7 shows the SPRi responses per marker, normalised by highest and lowest value. IgG1 was used as an isotype control to measure non‐specific binding. In addition, an empty spot without antibody was used as control as well.

Particles expressing lactadherin, flotillin‐1 and HSP70 were found in both EV fractions, since their responses exceeded the IgG1 response. In short, all three positive signals indicate the presence of membrane particles and thus EVs or similar particles. The typical EV markers, tetraspanins CD9, CD81, CD63, did not show elevated signals. It could be that the EVs showed more affinity towards the antibodies for lactadherin, flotillin‐1 and HSP70. For the contaminants albumin and ApoE, a clear difference could be observed between non‐purified CSF and both EV fractions, since these did not exceed IgG1 levels in the EV fractions while it did in crude CSF. This suggests that these proteins were successfully removed in the SmartSEC protocol.

### 3.5 Nanoparticle tracking analysis (NTA) and protein quantification

Particle concentration and size were measured by NTA(ZetaView) and protein concentration measured with the NanoOrange protein quantification kit.

**FIGURE 8:**
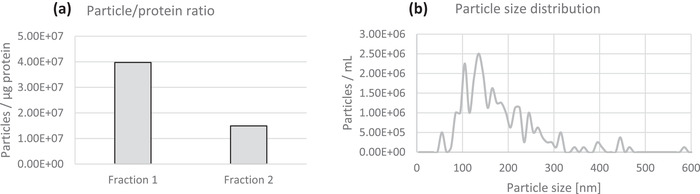
(a) Particle/protein ratio for fraction 1 and fraction 2, based on scatter mode nanoparticle tracking analysis and NanoOrange protein quantification, taking into account the dilution factor. (b) Particle size distribution for fraction 1, based on fluorescence mode nanoparticle tracking analysis

For the particle/protein ratio, ZetaView scatter mode results were used. Here, fraction 1 counted 126 particles per frame in average, resulting in a final concentration of 9.7×10^7^ particles per millilitre and a median particle size of 146.0 nm. For fraction 2, the average counted particles per frame was 52, resulting in a final concentration of 4.0×10^7^ particles per millilitre and a median particle size of 162.2 nm. For the particle size distribution, ZetaView fluorescence mode results were used from fraction 1 labelled with CMG, as the results from fraction 2 were below detection limit, meaning the number of counted particles per frame was below 50. For fraction 1, a concentration of 7.7×10^7^ particles per millilitre (dilution factor and correction factor taken into account) was observed, and a median size of 135.0 nm in a measurement where, in average, 76 particles were measured per frame. In conclusion, some particles without a lipid membrane (and thus non‐EVs particles) were excluded using CMG fluorescence, leading to a lower median size. The dilution factor was taken into account for both graphs in Figure 8 below.

With the NanoOrange protein quantification assay, an average result of 2.44 μg/ml was achieved for four replicates of fraction 1, and 2.68 μg/ml for four replicates of fraction 2. Taking into account the protein quantification results, 3.98×10^7^ particles/μg were measured in the first fraction, in contrast to 1.49×10^7^ particles/μg in the second fraction. This can indicate an increase in contamination proteins in fraction 2 by 2.67‐fold. Comparing both fractions, the protein content and particle concentration analysis suggested a higher particle yield and the particle/protein ratio suggested a greater apparent purity achieved in fraction 1 compared to fraction 2.

### 3.6 Liquid chromatography tandem mass spectrometry (LC‐MS/MS) analysis

**FIGURE 9:**
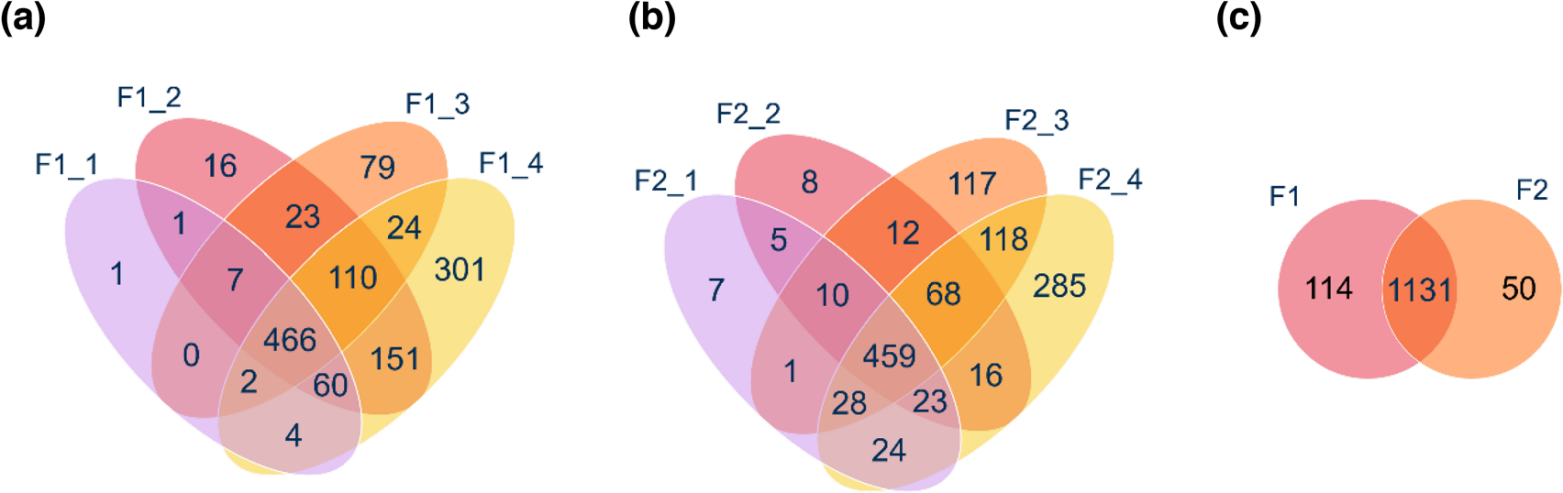
Venn diagrams showing (a) the number of overlapping proteins in the different replicates of fraction 1 (F1_1, F1_2, F1_3, F1_4), (b) the number of overlapping proteins in the different replicates of fraction 2 (F2_1, F2_2, F2_3, F2_4) and (c) the number of overlapping proteins in fraction 1 and fraction 2

LC‐MS/MS analysis was performed on fraction 1 and 2 of four separately isolated aliquots of CSF. In the first fraction. 541, 834, 711 and 1118 proteins were identified, respectively. In the second fraction, 557, 601, 813 and 1021 proteins were identified, respectively. In total, in the first fractions, 1245 different proteins were detected of which 397 proteins were detected in only one sample, 203 proteins in two samples, 179 in three samples and 466 proteins were identified in every replicate (Figure 9). In the second fractions, 1181 different proteins were detected of which 417 proteins were detected in only one sample, 176 proteins in two samples, 129 in three samples and 459 proteins were identified in every replicate (Figure 9). Thus, fraction 1 seemed to get slightly more reproducible results. An overview of all identified proteins is shown in the Supplementary data. A gene ontology (GO) enrichment was done using DAVID Bioinformatics Resources 6.8. Table 5 shows the top five of the GO enrichment of the identified proteins in fraction 1 against a CSF background (sorted by lowest *p*‐value), and Table 6 in fraction 2. Of the total number of 1245 identified proteins in the first fractions, 768 are extracellular exosome‐related according to the GO annotation. This results in 65.9% of the identified proteins. Of the 1181 identified proteins in the second fractions, 733 are extracellular exosome‐related according to the GO annotation. This results in 65.0% of the identified proteins. As the CSF background is already enriched in extracellular exosome proteins, relative to the total proteome background (40.7%), this results in an enrichment of 1.62 for fraction 1 and 1.60 for fraction 2 relative to CSF. The complete GO enrichment analyses of fraction 1, fraction 2 and the CSF proteome are shown in the supplementary data.

**TABLE 5:** Top five GO enrichment of fraction 1

Category	GO name	ID	p‐value
**GOTERM_CC_DIRECT**	Extracellular exosome	GO:0070062	3.7E − 95
**GOTERM_CC_DIRECT**	Blood microparticle	GO:0072562	4.1E − 33
**GOTERM_CC_DIRECT**	Extracellular matrix	GO:0031012	2.1E − 26
**GOTERM_CC_DIRECT**	Cytosol	GO:0005829	5.2E − 26
**GOTERM_BP_DIRECT**	Complement activation, classical pathway	GO:0006958	1.6E − 24

**TABLE 6:** Top five GO enrichment of fraction 2

Category	GO name	ID	p‐value
**GOTERM_CC_DIRECT**	Extracellular exosome	GO:0070062	3.3E − 85
**GOTERM_CC_DIRECT**	Blood microparticle	GO:0072562	4.4E − 34
**GOTERM_CC_DIRECT**	Extracellular matrix	GO:0031012	1.2E − 26
**GOTERM_BP_DIRECT**	Complement activation, classical pathway	GO:0006958	3.2E − 25
**GOTERM_CC_DIRECT**	Extracellular space	GO:0005615	2.0E − 19

In Table 7, the typical EV‐specific proteins, lipoprotein markers and the most abundant proteins in crude CSF are shown that could be detected. They are relatively quantified by comparing the average LFQ intensity of all four samples, for both fraction 1 and fraction 2. As seen in the table below, EV‐markers CD9, CD81, lactadherin, syntenin‐1 and syntenin‐2 were detected more in fraction 1 than fraction 2. For lipoprotein markers ApoA‐I, ApoA‐II, ApoB‐100, ApoC‐I, ApoC‐II, ApoC‐III and ApoE, the average LFQ intensity was higher in fraction 2. Furthermore, most of the abundant CSF proteins showed a higher LFQ intensity in fraction 1. Also several apolipoproteins are highly abundant in crude CSF. Albumin is seen as the highest abundant contaminant in CSF, and has a higher concentration in fraction 2. Most important here is that the abundant proteins do not suppress the detection of the EV markers, as can be the case due to limited dynamic range of LC‐MS. In this study, the abundant proteins seem depleted sufficiently. Also, proteins have been observed to interact with the surface of several nanoparticles, such as artificial nanoparticles and viruses, and form a protein corona. Recently, research suggests the existence of a protein EV corona as well. This would make the distinction between contamination proteins and inherent proteins more challenging. For example, a recent study on CSF found the major abundant proteins, like albumin, complement C3 and transthyretin to be part of the protein corona attached to artificial nanoparticles.

**TABLE 7:** Typical EV‐markers, lipoprotein markers and the most abundant proteins from CSF that were detected in this experiment by LC‐MS/MS

EV markers	LFQ intensity fractions 1	LFQ intensity fractions 2	Abundant CSF proteins	LFQ intensity fractions 1	LFQ intensity fractions 2
**CD9**	4,49E+05	1,57E+05	Albumin	3,44E+08	6,29E+08
**CD81**	8,16E+05	1,49E+05	α‐1‐antitrypsin	1,44E+07	3,15E+07
**Lactadherin**	7,93E+05	2,58E+05	α‐2‐macroglobulin	2,84E+07	1,99E+07
**Syntenin‐1**	5,66E+05	1,68E+05	Complement C3	3,42E+07	3,88E+07
**Syntenin‐2**	7,43E+03	0,00E+00	α‐1‐acid glycoprotein 1	2,52E+06	1,82E+06
**Lipoprotein markers**	**LFQ intensity fractions 1**	**LFQ intensity fractions 2**	α‐1‐acid glycoprotein 2	1,03E+06	7,82E+05
**ApoA‐I**	1,47E+07	3,04E+07	Haptoglobin	7,08E+06	6,86E+06
**ApoA‐II**	1,57E+06	3,10E+06	Transthyretin	1,06E+07	6,95E+06
**ApoB‐100**	3,54E+06	4,53E+06	Serotransferrin	3,42E+07	3,03E+07
**ApoC‐I**	3,81E+04	5,18E+04	Lactotransferrin	4,47E+06	2,60E+06
**ApoC‐II**	1,33E+04	2,29E+04			
**ApoC‐III**	1,84E+05	3,70E+05			
**ApoE**	1,16E+07	1,20E+07			

The average LFQ intensity of all four samples was taken, both for fraction 1 and for fraction 2.

It should be noted that the average of the LFQ intensities was looked at, and this same tendency could not be observed in each sample separately. This could be explained by the sample preparation protocol where two drying steps and an acetone precipitation without visible pellet are included. These steps possibly cause a lack of reproducibility.

## 4 DISCUSSION

Current published studies most often make use of pooled samples from multiple patients to isolate EVs from larger volumes of CSF, to overcome the EV concentration challenges. However, in order to perform a biomarker study in a patient cohort, it is important to take into account all individual patient information and thus to not pool patient samples. Multiplexed MS labels can only combine a dozen samples on average and are, therefore, not suited for large cohort studies. Moreover, it is valuable to be able to work with small volumes of CSF since biobanks often only have small volumes available of this biofluid. In this way, more samples can be collected from different patients, increasing the statistical power.

Furthermore, most used EV isolation methods work best for larger volumes of samples, which is why in this study, the SmartSEC HT EV isolation kit was evaluated for the isolation of EVs from 500 μL CSF starting volumes. This isolation results in two fractions of each 500 μL, which were compared to each other by use of various methods, to know which fraction yielded most EVs with the least contamination.

To determine the size, TEM, AF4‐MALS and (fluorescence) NTA were used. TEM showed slightly smaller particles than NTA, where also a difference between scatter mode and CMG fluorescence was observed. Such differences could be explained by the shrinkage of EVs on a TEM grid (VAN DER POL et al., 2014) and by the greater presence of contaminating particles, which are detected in scatter mode but not by CMG fluorescence. It should be noted that biological light scattering properties are quite low and one study comparing two NTA methods concluded that the ZetaView lower size detection limit was only 50 nm (BACHURSKI et al., 2019), excluding the smallest particles that could be observed by TEM. Furthermore, for fraction 2, the fluorescence signal was below detection limit, so these results could not be taken into account. Therefore, the ratio particles per microgram were calculated with scatter mode results and include all particles present in the samples, not only EVs. This ratio is regularly used as a sample purity check to exclude that there is a lot of protein contamination not coming from particles (WEBBER & CLAYTON, 2013). However, also non‐EV particles are taken into account in this ratio.

Sizes observed by AF4 are slightly larger than seen with TEM and NTA. Here, like in NTA scatter mode, all particles are measured by light scattering and no conclusions could be drawn with great certainty about the purity of the samples since the UV signal was below detection limit. However, the fact that this detection limit was not reached, does suggest that there was not much protein contamination in neither sample. We suspect that the differences observed in size are due to the small concentrations, scarcely passing the detection limit, providing a less accurate measurement.

By use of the previously mentioned methods, both EVs and other nanoparticles with a lipid membrane are measured. Exomeres, for example, are also considered intercellular communicators carrying cargoes between cells (ZHANG et al., 2019). As these cargoes could also contain proteins significant for biomarker research, they are not considered a contamination to be avoided. LDLs, on the other hand, LDLs are composed of approximately 75% lipid and 25% protein. Of these proteins, more than 95% are apolipoproteins (SEGREST et al., 2001). It is recommended to avoid lipoproteins in, for example, mass spectrometry, as they can interfere with biomarker analyses if the apolipoproteins are more abundant than the disease‐specific proteins. Especially since the presence of lipoproteins is influenced by, for instance, the diet (WOJCZYNSKI et al., 2011), and thus will cause interindividual variance.

With SPRi, the presence of lactadherin, flotillin‐1, and HSP70 in both fraction 1 and 2 suggests the presence of lipid membranes. As lactadherin binds to phosphatidylserine, SPRi lactadherin detection suggests the presence of outer leaflet phosphatidylserine. HSP70 is incorporated in EV membranes (and possibly exomeres). Flotillin‐1 is a membrane protein (MISEV, 2018). However, the signals of the three typical tetraspanins CD81, CD9 and CD63 did not exceed the IgG1 signal and thus were not detected. By use of ExoView, these three tetraspanins were the only antibodies used and all three were detected in fraction 1. As was also the case for crude CSF, capture antibodies CD81 and CD9 resulted in the highest signal of the three fluorescent antibodies used. Furthermore, a trade‐off could be observed where the CD9‐captured EVs showed a higher CD81 signal and the other way around. This could mean that most of the isolated EVs in fraction 1 only have one epitope per particle. It could be that in fraction 2, and its lack of tetraspanin signal, the particles detected in the other methods are non‐EV lipid particles. Another possible explanation is that fraction 2 consists of even smaller EVs (as observed with AF4‐MALS), causing steric hindrance for two antibodies to bind on one particle (as briefly mentioned in ALLELEIN et al. (2021)). Moreover, fraction 2 is eluted with the SmartSEC buffer, possibly interfering with one of the characterisation methods used. The fact that these tetraspanins were detected by ExoView and not by SPRi could be explained if the EVs had a greater affinity for the antibodies of lactadherin, flotillin‐1 and HSP70, which competed with the EV binding on CD81, CD9 and CD63 antibodies. This would be a logical result if the EVs have less tetraspanins in their membrane than lactadherin, flotillin‐1 and HSP70. It should be noted that crude CSF did show an elevated CD81 signal, but since cells are still present here, this could come from the plasma membrane instead of EVs . Two other SPRi positive signals in crude CSF indicated the abundant presence of albumin and ApoE, which were clearly depleted in both EV fractions. This shows an effective removal of abundant contaminating proteins, which would certainly interfere with the biomarker analyses. The removal of some abundant proteins was also observed by LC‐MS/MS, as typical EV markers were detected and thus not masked by other proteins in the sample. Furthermore by LC‐MS/MS, EV‐related proteins were observed to be enriched in both fractions, and LFQ of these proteins showed higher intensities in fraction 1 than in fraction 2.

Of the papers mentioned in Table 2, only three papers looked at isolations starting from less than 1‐ml CSF (GUHA et al., 2019; NORMAN et al., 2021; PIERAGOSTINO et al., 2019). Of these, only GUHA et al. (2019) mention characterisation methods for EV validation. After EV isolation by immunodepletion and ExoQuick, an average diameter of CSF EVs of 126 nm was measured with a ZetaView instrument, and concentration of 1.9×10^11^ particles per millilitre in average. In our study, both fraction 1 and fraction 2 (respectively 146.0 and 162.2 nm) were observed to be slightly bigger and less numerous (relatively 9.7×10^7^ particles per millilitre and 4.0×10^7^ particles per millilitre). They do not mention the purity of the sample, and it is probable that their samples contain more contaminating particles because of the polymer precipitation performed with ExoQuick.

Additional experiments could be performed to obtain more information about the CSF EV fractions, for example, to identify the small nanoparticles observed by TEM. Since, for example, albumin was shown present by LC‐MS/MS in both fraction 1 and 2, these results could be compared to those of non‐purified CSF to check how much of the abundant proteins were depleted, as was now done with the less‐sensitive SPRi. Additional experiments could also point out if the SmartSEC buffer influences the results of fraction 2, or alternatives can be searched to elute this fraction with. To study the efficiency of the SmartSEC isolation, a recombinant EV sample could be used, where the number of EVs loaded onto the column could be compared to the number of EVs eluted. Finally, we recommend comparing different CSF input volumes, as the kit is optimised for 250–500 μL of plasma/serum samples.

We have submitted all relevant data of our experiments to the EV‐TRACK knowledgebase (EV‐TRACK ID: EV210252) (VAN DEUN et al., 2017). The EV‐METRIC score is 71 %.

## 5 CONCLUSION

In conclusion, characterising EVs from smaller volumes of CSF is challenging because of a lower abundance and the inability to reach the detection limit of the characterisation methods. Nevertheless, we were able to characterise both EV fractions eluted from the SmartSEC HT EV isolation kit by use of various complementary methods. EVs were observed and characterised both visually, immunologically, and by means of LC‐MS/MS. We believe that in both fractions, EVs were eluted, but that the concentration in fraction 1 was higher, while retrieving a better purity. Results for fraction 1 seemed to be consistent across all different characterisation methods. Additionally, EVs could be observed with confidence. For fraction 2, it is less clear whether non‐EV particles were equally observed and/or, perhaps, a smaller kind of EV.

By and large, we showed that our complementary methods only necessitated a mere 500 μL of CSF starting volume, and, conclude that fraction 1 consisted of sufficiently pure EVs for further biomarker studies. This means that future EV extractions may be based upon smaller CSF quantities, such as from individual patients. In that way, patient samples do not have to be pooled and individual patient information can be included in forthcoming studies, potentially linking EV content, size and distribution to individualised neurological diagnoses.

## CONFLICT OF INTEREST

The authors declare no conflict of interest.

## Supporting information

Supplementary information
